# Dimorphic Fungal Infections in HIV/AIDS Patients with non-TB Chronic Cough at Mulago Hospital, Kampala, Uganda

**DOI:** 10.21203/rs.3.rs-3194828/v1

**Published:** 2023-07-25

**Authors:** Prossy Kiconco, Beatrice Achan, Irene Najjingo, Moses Sanya, Alfred Okeng, Winnie Binoga, Benson Musinguzi, Freddie Bwanga

**Affiliations:** Makerere University; Makerere University; MBN Clinical Laboratories; MBN Clinical Laboratories; MBN Clinical Laboratories; MBN Clinical Laboratories; Muni University; Makerere University

**Keywords:** Dimorphic, Fungal, TB, Chronic, Cough, HIV/AIDS, Pulmonary, Opportunistic

## Abstract

**Introduction:**

Dimorphic fungi cause infection following inhalation of spores into the pulmonary system. In the lower respiratory tract, the conidia transform into the yeast phase which are engulfed by alveolar macrophages and may be destroyed without disease manifestation. However, in some cases they may persist and cause fungal disease characterized by formation of granulomas in the infected tissues, which may mimic MTB.

**Objective:**

To explore if dimorphic fungi play any role in pulmonary disease among XpertTB/RIF Negative HIV Patients with chronic cough attending ISS Clinic at Mulago hospital Uganda.

**Methods:**

Sputum samples were collected from 175 consented HIV infected patients attending ISS Clinic. Upon Xpert/RIF test at ISS Clinic 21 of these tested positive, the 154 negative sputum samples were then subjected to PCR for dimorphic fungi at MBN Clinical Laboratories. Singleplex PCR using specific primers was used to detect a target sequency in the gene of each dimorphic fungi of interest, the resulting amplicons were electrophoresed on a 2% gel then visualized under UV light.

**Results:**

*Blastomyces dermatitidis* and *Tarolomyces marneffei* were detected in 16.4% of the studied participants, with 9.1% and 7.1% respectively and 83.8% of the participant sample had no dimorphic fungi. Coccidiodes immitis, *Paracoccidiodes brasiliensis* and *Histoplasma capsulatum* were not detected in any of the participants

**Conclusion:**

Dimorphic fungi play a role in pulmonary disease among the HIV/AIDS with non- TB chronic in Uganda.

## Introduction

Pulmonary infections cause the highest cases of morbidity and mortality in HIV-infected individuals(1–3).The clinical presentation of HIV/AIDS and the occurrence of opportunistic infections depend on different factors such as the presence of endemic diseases, quality of health services, availability of and access to antiretroviral treatment and levels of education of the population (3). While Dimorphic fungi are considered opportunistic pathogens, their contribution in causation of pulmonary disease among immunocompromised patients with non-TB chronic cough in Uganda is poorly understood. However, studies done elsewhere have reported different findings. A clinical peer reviewed report in 2007 in Michigan showed increased incidence of pulmonary Histoplasma capsulatum as a self-limiting illness occurring mostly in children exposed to the organism for the first time, however, the study did not involve immunocompromised individuals (4). A study done in 2013 in South Africa among 13 HIV positive patients with disseminated fungal infection found 24 cases of Dimorphic fungal infections 13 of which were caused by Emmonsia spp and these infections clinically mimicked tuberculosis on chest x-ray, however low numbers were investigated (5, 6). Furthermore a medical review at the University of California in 2011 stated that the incidence of coccidioidomycosis an example of these dimorphic fungi continues to rise and primary coccidioidal pneumonia accounts for 17–29% of all cases of community acquired pneumonia in endemic regions, however this only considered region far away from the Sub Saharan African (7). In Ugandan settings studies on pathogens that cause disease in immunocompromised have focused mainly on MTB, *Pneumocystis jirovencii* and *Cryptococcus neoformans* (8, 9). This clearly showed that the role of dimorphic fungi in causation of pulmonary disease in immunocompromised patients presenting with non-TB chronic cough in Uganda was not well understood yet these fungi have similar immune response and may present with symptoms similar to pulmonary TB. The goal this study was to explore whether dimorphic fungi play a role in causation of chronic cough among immunocompromised patients presenting with non-TB chronic cough at Mulago hospital. And if patients were found to have these pathogens our results would have great impact toward the policy on care of immunocompromised patients who present with non-TB chronic cough.

## Materials and Methods

This was a cross-sectional study conducted from July 2020 – Feb-2021 which involved Patients who came to attend the Mulago ISS clinic. The case definition in this study was: An XpertTB/RIF Negative HIV-infected Patient who was presenting with chronic cough at the Mulago hospital ISS clinic. The main study group consisted of adult participants (≥ 18 years of age) who were infected with HIV and were presenting with chronic cough. The participants were consecutively sampled; here every patient who reported to ISS clinic and met the criteria was included in the study.

Other inclusion criteria were giving written informed consent to both provide a sputum sample and participate in the study. Participants were excluded from the study if they turned XpertTB/RIF positive, if they refused to consent and if they failed to provide a sputum sample or had other serious medical hindered them.

## Data collection

Pre-tested questionaries and case report forms were used to record socio demographic and clinical data from the study patients. Furthermore every specimen was accessioned and given unique codes at the Laboratory. These laboratory forms were then be used to record data obtained from tested the specimens.

### Specimen collection, packaging and transport.

Sputum samples were collected from 175 participants who consented to take part in the study. They were provided with two wide mouthed sputum containers with a screw cap where they were instructed to cough spontaneous in open air and collect about 3mls of sputum in each sputum container. One for Xpert TB/RIF test at ISS clinic and the other for dimorphic fungal PCR. Sample containers with sputum were assigned unique identifiers (study numbers) to represent each participant sample. Samples were then packaged based on triple packaging system where sputum containers were tightened to prevent spillage; enough cotton was then wrapped to absorb the sample in case of a spillage. The samples were then put in a biohazard bag; the bag sealed off and then placed in a cool box. The cool box was properly sealed and transported in an upright position to the Laboratory.

#### Laboratory procedures.

At the ISS clinic, Xpert TB/RIF test was done on one of the sputum samples collected, the positive samples from here were excluded from those to be run for dimorphic fungi at MBN laboratories. The sputum samples that turned negative were included in our study.

#### Sputum processing.

Sputum specimens were mucolysed by adding of *N*-acetyl-*L*-Cysteine (NALC)-4% Sodium Hydroxide (NaOH) which digests and decontaminates the sputum specimens. The sputum specimens were diluted with sterile phosphate buffer, pH 6.8. The mixtures were then centrifuged at 3000 rpm for 15 min and the sediments re-suspended in 1 ml of sterile phosphate buffer, the mixture was vortexed to homogenize. The mixture was then transferred into a 1.5 mls microtube and centrifuged at a speed of 14000 rpm for 10 minutes, the supernatant was aliquoted and the obtained pellet mixture with a 100 uL of PCR water, vortexed to mix.

#### DNA extraction.

DNA was extracted using boiling method, the processed sputum sample capped in microtube was put on a heat block at 100 for 30 minutes, and the sample was then put in a sonicator for 15 minutes, centrifuged and the supernatant used as DNA.

#### Primer and primer dilution.

The primers were procured in lyophilised state, they were then prepared and diluted as shown in the Table 1

#### Preparation Pre-PCR mix.

For each primer target reagent were prepared as detailed in Table 2

#### DNA amplification.

We performed Singleplex PCR where we targeted a single dimorphic fungi gene at ago. The amplification of the Fungal DNA was done using GTQ-CYCLER 96 thermocycler machine (Hain Life science GmbH, Nehren, Germany). See programs in Table 4.

#### Agarose gel electrophoresis.

Agarose gel electrophoresis was performed by dissolving 4g of agarose powder into 200mls of TAE buffer, the mixture was heated for 5 minutes, and left to cool for some time, ethidium bromide was then added and the warm mixture poured on a casting tray and then left to set at room temperature. To the amplified DNA 5 μl of the loading dye (BioLabs New England) was added, 15ul of the mixture was then loaded on the gel and ran at 120V on a 4% Agarose gel for electrophoresis.

#### Interpretation.

**Figure 2:**
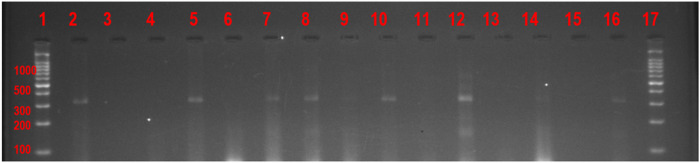
- Agarose gel electrophoresis image (Gel-04022021–2) showing the 363 bp DNA amplicons band of the *BAD*1 gene fragment of *Blastomyces dermatitidis*. Lanes 1 and 17 = 100bpb DNA ladder, Lane 2 = Positive Control (ATCC 26199) Lane 3 = Negative control (PCR Water), Lanes 4, 6, 9,11,13,14, and 15 = Clinical samples negative for *Blastomyces dermatitidis*. Lanes 5,7,8,10,12 & 16 = Clinical samples positive for *Blastomyces dermatitidis*

**Figure 3:**
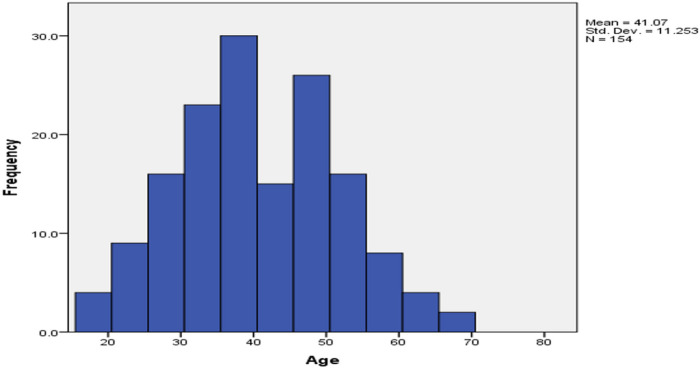
Age distribution of the study participants

Visualization and analysis were done under UV light. Positive reactions for *Tarolomyces marneffei* and *Blastomyces dermatitidis* yielded amplicons of 927 bp and 363 Pb respectively which were viewed on an agarose gel after electrophoresis. Positive controls and negative controls were run alongside clinical samples during the electrophoresis as detailed in Figs. 2 and 3.

#### Quality assurance.

We used a qualified assistant nurse on ground to assist in sample collection, we ensured that the samples were labelled well to avoid mix up. While running the PCR, a sample containing known positive Dimorphic Fungal DNA detailed in Table 4 was always run along as positive quality control and a sample containing PCR water as the template was always used as a negative control. These control strains were handled in a class two biosafety cabinet to ensure safety.

### Data management and analysis.

All case report forms and consent forms were examined for completion and results obtained were verified for any clerical errors by comparing the final lab report results with the raw data generated in the lab that details every step undertaken before concluding on the results to be reported. After the verification process, data was coded and entered in the system software and extracted as an excel sheet into a predesigned template in Microsoft excel and then exported to SPSS software for analysis. We automatically generated an excel sheet from the MBN Laboratory Information System and then exported the data to SPSS (Statistical Package for the Social Sciences software) and STATA for analysis. For SPSS we grouped the data into Categorical and numerical data. Categorical data included gender, district of origin, occupation, travel history, underlying condition and age group. Categorical data was analysed and the outcome presented as percentages, frequencies and proportions in tables and graphs. Numerical data included age and this was analysed and presented as mean, mode, and interquartile range.

For STATA bivariate analysis to assess the factors associated with dimorphic fungal infections, we grouped data into dependent and independent variables, dependent variables included age, gender, district of origin, occupation, travel history, underlying condition and age group, independent variables were our outcomes. We then used logistic regression model since the prevalence of each individual fungi is less than 10%. We assessed for significancy of association using *p-value* and confidence interval. *P-value* below 0.05 was considered significant.

### Ethics Consideration.

The study was approved by Makerere University Higher Degrees Research and Ethics Committee, School of Biomedical Sciences (SBSHDREC). The study also obtained administrative permission at Mulago National Referral Hospital ISS clinic. Participants were requested to consent and confidentiality was maintained at all times. The participants were free to withdraw from the study at any time which would not affect their clinical or health care.

## Results

### Description of the studied population

**Figure 4:**
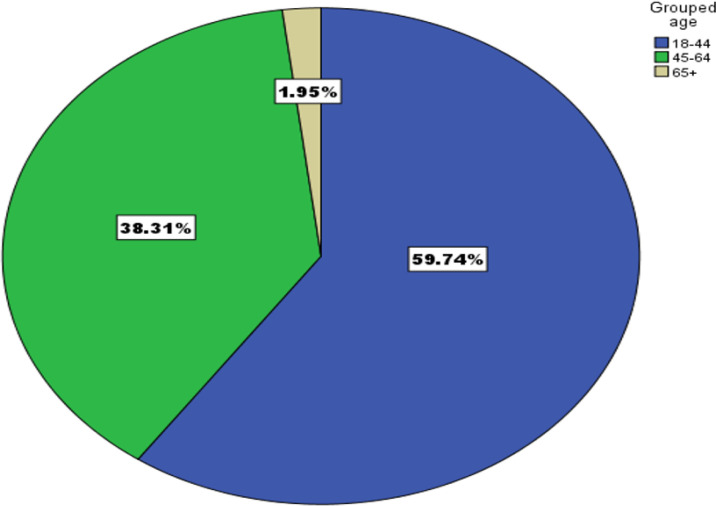
Age groups of the study participants

One hundred and seventy-five HIV/AIDS patients were sampled consecutively and tested for XPERT-MTB/RIF, of these one hundred and fifty-four who turned negative were enrolled in this study. 84 (54.5%) of these were females, all were new TB suspects with no retreatment cases. The participants reported to be mostly from central Uganda with 107 (74%) from Kampala and Wakiso districts, all expect one had not travel out of the country in the last one year. The studied participants reported to be majorly farmers and small business holders (60% and 47% respectively. (Details in Table 6). The minimum, maximum, mean and median age of the participants were 18, 81, 41.07 and 40 years respectively with 60% under the age group of 18–44 (refer to Figs. 3 and 4). The age of the participants was normally distributed.

#### Proportion of participants with dimorphic fungal infection.

Of the 154 participants studied 14(9.1%) were positive for *Blastomyces Dermatitidis*, 11(7.1%) were positive for *Tarolomyces Marneffei*, while none of the participants was positive for *Histoplasma capsulatum Coccidiodes emmitis* and *Paracoccidiodes brasiliensis* as detailed in Table 7)

### Distribution of fungi versus different demographic factors

Factors that were assessed include gender, age, occupation, travel history, district of residence and any other underlying condition other than HIV/AIDS. *Blastomyces dermatitidis* was more prevalent in males while *Tarolomyces marneffei* was more prevalent in females. The most affected age group was (18–44) and none of them had any other underlying condition. Majority of the cases were Kampala residents who are majorly business people and none of them reported to have travelled out of the country.

#### Bivariate analysis of factors associated with having Blastomyces dermatitidis and Tararomyces marneffei

From bivariate analysis below, several factors used to assess for prevalence of dimorphic fungi were found to differ among the participants. These factors were included in the model to find out what association each of these has with the outcome. All the factors were not associated with the outcome (Blastomyces *dermatitidis* and *dermatitidis*) apart from gender which showed independent association but was not considered statistically significant because of the small sample size. Prevalence of Blastomyces dermatitidis

## DISCUSION

This study aimed at exploring the role played by dimorphic fungi in pulmonary disease among HIV/AIDS patients with chronic cough at Mulago Hospital Uganda. Out of the participants who turned up at the clinic with symptoms of pulmonary disease 21 of these were XpertTB/RIF positive, the rest 154 were negative for XpertTB/RIF (these were our study participants).

Our study found out that indeed dimorphic fungi play a role in pulmonary disease, we found *Blastomyces dermatitis* and *Tarolomyces marneffei* in 14(9.1%) and 11(7.1%) participants respectively this could imply that out every 100 patients who turn out to be MTB Genexpert negative 10 of these dimorphic fungi go undiagnosed. Our study found out that none of the participants who had Blastomyces dermatitidis had travelled abroad to the countries where these dimorphic fungi are highly endemic(15), this means that these dimorphic infections do exist in our Ugandan setting though in most case they go undiagnosed. Our study also found that most of the people who were infected by Blastomyces dermatitidis were doing outdoor businesses and farming with none doing indoor business, this is in agreement with the study that was done to assess the risk factors for acquisition of endemic Blastomycosis in Canada where they found out *that* a significantly greater proportion of patients with Blastomycosis than control subjects were involved in outdoor occupations (16).In this study we found out that males were infected with *Blastomyces dermatitidis* more than females, this was the same finding as another study that was conducted in regions of North America that border the Great Lakes and the St. Lawrence River in 2010, this finding was attributed to the fact that males are more likely to participate in activities that put them at risk for exposure to *Blastomyces dermatitidis* (17).

*Another finding in our study was that Tarolomyces marneffei formally known as Penicillium marneffei which accounted for 7.1% of these dimorphic fungal infections in our participants and out of these 72% were females while 28% were males, this is different from the findings of the study done in* Vietnam, between July 2006 and September 2009 where the majority of those infected were males(18).

Majority of our study participants who turned out positive for dimorphic fungi were from urban centers like Kampala and Mukono, these places are mostly dusty and busy and this can be compared to the high prevalence in other big countries like New York and Canada where activities like excavation are done(19). Out the 154 participants 129 were negative for all the dimorphic fungi but were still presenting with symptoms of pulmonary disease, these could be accounted for by some other pathogens like fungi and bacteria, a study that looked at Aetiology of Pulmonary Symptoms in HIV-Infected Smear Negative Recurrent PTB Suspects in Kampala, Uganda by Okwera and Bwanga in 2011 found *Pneumocystis jirovenci* in 6.7% and 28.3% had bacteria(9). This implies that some of the participants were negative for dimorphic fungi in our study were probably infected by either of these. *Histoplasma capsulatum*, *Paracoccidiodes brasiliensis* and *Coccidiodes immitis* were not isolated in any of the participants we studied.

Contrary to our study findings where no *Histoplasma capsulatum* cases were found in sputum of HIV/AIDS patients at mulago, a study was done in similar setting assessing for antibodies to *Histoplasma Capsulatum* in HIV-infected persons with suspected meningitis and 1.3% of these were found to be antibody IgG positive. A clinical report published in 2007 in Michigan showed increased incidence of pulmonary Histoplasma capsulatum as a self-limiting illness occurring mostly in children exposed to the organism for the first time(4), in our setting we used only adults and this could have accounted for the different findings. *A M*edical review at the University of California in 2011 assessing dimorphic fungi stated that the incidence of coccidioides immitis infections was high and that primary coccidioidal pneumonia accounted for 17–29% of all cases of community acquired pneumonia in endemic regions, however this is not in line with our findings because in our study Coccidiodes immitis was not detected(7).

All our participants had no underlying conditions other than HIV/AIDS and this was the same as the study that was done non endemic areas of China. A study done in 2013 in South Africa among 13 HIV positive patients with disseminated fungal infection found 24 cases of Dimorphic fungal infections 13 of which were caused by *Emmonsia* spp, our study did not look out for this fungi(20).

These fungi are not routinely investigated in our facilities when MTB Genexpert is negative, we found out patients are put on antibiotics and it’s unclear whether they all get well or not. In our study participants were not followed up so we can’t know whether these peoples infection cleared. Although the method we used may not be affordable by the general population, still something can be done in these facilities because Genexpert uses the same technique PCR. Since the facilities managed to afford these Genexpert machines, effort should put to assist referral facilities to test for these fungi so as to effectively manage these patients with pulmonary disease.

## CONCLUSION

This study has shown that dimorphic fungi play a role in pulmonary disease among the HIV/AIDS with non-TB chronic in Uganda. Since none of the study participants had travelled abroad, this implies that these dimorphic fungal infections do exist in our Ugandan setting but they are not usually looked out for in Ugandan settings

## RECOMMENDATION

We recommend patients of chronic cough who are TB negative to be investigated for dimorphic fungal infections as well.

## Figures and Tables

**Figure 1:**
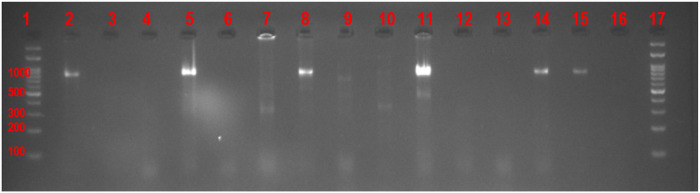
- Agarose gel electrophoresis image (Gel-01022021–1) showing the 927 bp DNA amplicons band of the *mef2* gene of *Tarolomyces marneffei* (formally called *Penicillium marneffei*). Lanes 1 and 17 = 100bpb DNA ladder, Lane 2 = Positive Control (ATCC 18224) Lane 3 = Negative control (PCR Water), Lanes 4,6,7,9,10,12,13 and 16 = Clinical samples negative for *Tarolomyces marneffei*. Lanes 5, 8, 11, 14 & 15 = Clinical samples positive for *Tarolomyces marneffei*

**Table 1:** Primers and primer dilution for PCR

	Primer name (Original concentration in mg)	Conc. / μg (X 1000)	PCR water added in uL	Final primer concn(1μg/μg)
*Blastomyces dermatitidis*	BLASTO - 1 (024)	0.24 x1000 = 240	240	1
	BLASTO - 2 (0.2)	0.20 x1000 = 200	200	1
*Tararomyces marneffei*	PMI-F (0.21)	0.21 x 1000 = 210	210	1
	PMI-R (0.18)	0.18 x1000 = 180	180	1
*Paracoccidiodes brasiliensis*	P.BLAS- F (0.31)	0.31 x1000 = 310	310	1
	P.BLAS- F (0.22)	0.22 x 1000 = 220	220	1
*Coccidiodes immitis*	COCI- F (0.33)	0.33 x 1000 = 330	330	1
	COCI- R (0.2)	0.20 x 1000 = 200	200	1
*Histoplasma capsulatum*	HIST-Msp1-F (0.2)	0.2 x 1000 = 200	240	1
	HIST-Msp1-R (0.29)	0.29 x 1000 = 290	200	1

**Table 2:** Preparation of the Pre-PCR mixture.

Master mix (QUIGEN cat no.201443)	Forward primer *	Reverse primer*	PCR buffer	Extracted DNA sample	Total volume
mg2+, DNTPS, Taq polymerase, reaction buffer					

**25 uL**	**1.5 uL**	**1.5 Ul**	**1 uL**	**5 uL**	**34 uL**

**Table 3:** Primer sets used for amplifying dimorphic fungi unique sequences.

Fungi	PRIMER SEQUENCE	TARGET GENE/PROTEIN	AMPLICON SIZE(bp)	Reference
*Blastomyces dermatitidis*	5’-AAGTGGCTGGGTAGTTATACGCTAC-3 5’-TAGGTTGCTGATTCCATAAGTCAGG-3	*badi* virulence gene promoter	363	(10)
*Tararomyces marneffei*	(5’TCGCCGGGGGACGTTTGT-3’)	*mef2, gene*	927	(11)
	(5’ ATGGTGGTGACCAACCCCCGCA3’)						
*H.capsulatum*	5’ ACA AGA GAC GAC GGT AGC TTC ACG 5’ GCG TTG GGG ATC AAG CGA TGA GCC	*mspTF msplR*	lll	(12)
*P.brasiliensis*	5’ ATGAATTTTAGTTCTCTTAACCTGGCTCTT 5’ CCTGCATCCACCATACTTCCTAGCCCA	*gp43–1 gp43–2*	1303	(13)
*Coccidioides immitis*	5’ AAG TTC TCA CTC CTC AGC GCT ATCG 3’ 5.ACA TTA AGG TTC CTC CCC TTC AAC C 3	csa gene sequence	520	(14)

**Table 4:** PCR conditions used for the different dimorphic fungi.

Fungi	Denaturation	Annealing			Elongation	References
*Blastomyces dermatitidis*	94 ^0^C for 5 minutes	94^0^C for 30 seconds	53 ^0^C for 30 seconds	72 ^0^Cfor 3.5minutes	72 ^0^C for 10 minutes	(10)
*Tararomyces marneffei*	95 ^0^C for 5 minutes	95^0^C for 30 seconds	55 ^0^C for 30 seconds	72 ^0^C for 2 minutes	72 ^0^C for 10 minutes	(11)
*H. capsulatum*	95 ^0^C for 5 minutes	95^0^C for 1 min	70 ^0^C for 1 min	72 ^0^C for 1 minutes	72 ^0^C for 5 minutes	(12)
*P. brasiliensis*	95 ^0^C for 5 minutes	95^0^C for 30 seconds	55 ^0^C for 30 seconds	72 ^0^C for 2 minutes	72 ^0^C for 1 minutes	(13)
*Coccidioides immitis*	94 ^0^C for 5 minutes	94^0^C for 30 seconds	50 ^0^C for 30 seconds	72 ^0^C for 2 minutes	5 ^0^C for 10 minutes	(14)

**Table 4:** ATCC strains used for different fungi

Dimorphic fungi	Control strain
*Tararomyces marneffei*	ATCC 18224
*Blastomyces dermatitidis*	ATCC 26199
*H. capsulatum*	ATCC 26032
*Coccidioides immitis*	ATCC 34021
*P. brasiliensis*	ATCC 200443

**Table 6:** Demographics of the participants

Gender		
Male		84(54.5%)
Female		70(45.5%)
**AGE**		
Minimum age		18 years
Maximum age		81 years
Age group 18–44 years		92(59.7%)
Age group 45–64 years		59(38.3%)
Age group ≥ 65 years		3(1.9%)
Median age		40
Mean age		41.07
Std. Deviation		11.253
Age Percentiles	25	32.00
	50	40.00
	75	49.00
Age interquartile range		17
**DISTRICT OF RESIDENCE**		
Kampala		67(43.5%)
Wakiso		47(30.5%)
Others		40 (25.97%)
**OCCUPATION**		
Farming		60(39%)
Business unclassified		47(30.5%)
Transport		11(7.1%)
Others		36 (23.4%)
**TRAVEL HISTORY**		
None		1 53(99.4%)
Sudan		1(0.6)
**MTB PARTICIPANT STATUS**		
**Gender**		
All new suspects		154(100%)
**OTHER UNDERLYING CONDITIONS**		
None		153(99.4%)
Diabetes		1 (0.6%)
**CLINICAL SYMPTOMS**		
Productive sputum		151 (98%)
Evening fever		105 (68.2%)
Night sweat		88 (57.1%)
Blood in sputum		3 (1.94%)

**Table 7:** Prevalence of dimorphic fungal infections

	Positive	Negative	Total
***Blastomyces dermatitidis***	**14 (9.1%)**	**140 (90.9%)**	**154 (100%)**
***Tarolomyces Marneffei***	**11 (7.1% )**	**143 ( 92.9%)**	**154 (100%)**
**Histoplasma capsulatum**	0 (0.00%)	154 (100%)	154 (100%)
**Coccidiodes emmitis**	0 (0.00%)	154 (100%)	154 (100%)
**Paracoccidiodes brasiliensis**	0 (0.00%	154 (100%)	154 (100%)

**Table 7:** Distribution of Dimorphic fungi (n = 25) versus different demographic factors

	*Blastomyces dermatitidis* (n = 14)			Penecillium marneffei (n = 11		
	Count	(95% CI)	*p-value*	Count	Odds ratio (95% CI)	*p-value*
**Gender**						
Males	11	1.0		3	1.0	
Females	3	(0.81–1.12)	0.075	8	3.5(0.86–12.24)	0.082
**Age**						
		(0.97–1.06)	0.583		0.97(0.91–1.02)	0.246
**Occupation**						
Others	**4**	**1.0**		**4**	**1.0**	
Business	6	(−0.49–1.97)	0.240	6	2.78(0.72–10.77)	0.138
Farming	4	(0.37–2.62)	0.140	1	1.37(0.14–13.17)	0.785
**District of residency**						
Central	13	(0.19–12.78)	0.679	11	-	-
Others	1	1.0	0		-	-
**Clinical symptoms**						
Productive cough	14	(0.05–0.14)	0.583	11	(0.03–0.11)	0.630
Evening fever	10	(0.04–01 5)	0.786	9	(0.03–014)	0.317
Night sweat	9	(0.04–017)	0.574	10	(0.05–0.18)	0.019
Blood in sputum	1	(− 1.1–1.77)	0.142	0	(0.03–0.11)	0.630
